# Drivers of Variation in the Optimal Spatial Structure of Collective Information Gatherers

**DOI:** 10.1007/s11538-026-01666-8

**Published:** 2026-06-10

**Authors:** Ross S. Walker, Gabriel Ramos-Fernandez, Denis Boyer, Sandra E. Smith-Aguilar, Xander O’Neill, Matthew J. Silk

**Affiliations:** 1 Department of Mathematics and Maxwell Institute for Mathematical Sciences, Heriot-Watt University, Edinburgh, EH9 3FL United Kingdom; 2 Institute of Ecology and Evolution, University of Edinburgh, Edinburgh, EH9 3FL United Kingdom; 3 Institute for Research on Applied Mathematics and Systems, National Autonomous University of Mexico, Mexico City, 04510 Mexico; 4 Global Research Centre for Diverse Intelligences, University of St. Andrews, St. Andrews, KY16 9AJ United Kingdom; 5 Institute of Physics, National Autonomous University of Mexico, Mexico City, 04510 Mexico

**Keywords:** Collective intelligence, Spatial structure, Social foraging, Space use, Swarm robotics

## Abstract

Collective systems that self-organise to maximise the group’s ability to collect and distribute information can be successful in environments with high spatial and temporal variation. Such organisations are abundant in nature, as sharing information is a key benefit of many biological collective systems, and have been influential in the design of many artificial collectives such as swarm robotics. Understanding how these systems may be spatially distributed to optimise their collective potential is therefore of importance in both ecology and in collective systems design. Here, we develop a mathematical model which uses an optimisation framework to determine the higher-order spatial structure of a collective that optimises group-level knowledge transfer. The domain of the objective function is a set of weighted hypergraphs, which can fully represent the spatial structure from a topological perspective. By varying the parameters within the objective function and the constraints, we determine how the optimal spatial structure may vary when individuals differ in their information gathering ability and how this variation differs in the context of resource constraints. Our key findings are that the amount of resources in the environment can lead to specific subgroup sizes being optimal for the group as a whole when individuals are homogeneous in their information gathering abilities. Further, when there is variation in information gathering abilities, our model implies that the sharing of space between smaller subgroups of the population, rather than the whole population, is optimal for collective knowledge sharing. Our results have applications across diverse contexts from behavioural ecology to bio-inspired collective systems design.

## Introduction

Diverse natural and artificial systems are organised to optimise the transfer and distribution of information across a group of individuals. Collectively intelligent systems such as robot swarms, characterised by a lack of central control, simple movement rules and local interaction patterns, are increasingly used across multiple complex applications (Schranz et al. 2020), such as environmental monitoring (Duarte et al. 2016), targeted drug delivery (Banharnsakun et al. 2016) and space exploration (Escoubet et al. 2001). Many of these artificial systems, designed for collection and distribution of information in complex, heterogeneous environments, are directly inspired by animal systems (such as eusocial insects; Krieger et al. (2000); Schranz et al. (2020); Banharnsakun et al. (2016)).

In these animal systems, information sharing is one of the key drivers of group-living (Evans et al. 2016; Brown et al. 1990). While group-living comes with a variety of associated costs, such as increased competition for resources (Alexander 1974) and risk of pathogen transmission (Romano et al. 2020; Sah et al. 2018), these costs are mitigated through the many benefits of group-living (Uetz et al. 2002; Salguero-Gómez 2024; Ebensperger et al. 2016), one of which is the sharing of foraging information (Brown 1986; Ebensperger and Hayes 2016). This is an especially significant benefit of group-living for species living in highly heterogeneous environments with a high degree of spatial and temporal variation in the quality of feeding sites (Bhattacharya and Vicsek 2014; Dunbar 1988; Clark and Mangel 1984; Kummer 2017; Moussaid et al. 2009; Ebensperger and Hayes 2016) (e.g. tropical forests for frugivorous species (Smith-Aguilar et al. 2016)). In such systems, the pooling of knowledge between conspecifics facilitates a more complete tracking of the current foraging environment than what individuals could manage on their own (Moussaid et al. 2009; Bonabeau et al. 1999; Papageorgiou et al. 2024).

Information sharing between individuals in a collective system can take a variety of forms. In swarm robotics, for example, individuals transmit their current knowledge through short-distance signals (Schranz et al. 2020). In animal systems, information sharing can take place in the form of vocalisations (e.g. in meerkats (*Suricata suricatta*); Gall and Manser (2017)), observation (e.g. in guppies (*Poecilia reticulata*); Laland and Williams (1997)) or following (e.g. in hooded crows (*Corvus corone cornix*); Sonerud et al. (2001)), and can be either active/intentional or passive/involuntary. In each of these cases, information transfer could potentially be costly to individuals in the short-term due to resource sharing, but this cost might be tolerated for future reciprocity (Wilkinson 1984; Carter and Wilkinson 2013) (depending upon total resource availability, which may determine the effective benefit of less cooperative strategies). There are two key commonalities across all of these forms of sharing. Firstly, the sharing can be considered a *higher-order* behaviour, meaning it does not only take place between dyads but also simultaneously between groups of 3 or more individuals (Silk et al. 2022; Betti et al. 2025). Recent modelling studies related to information transfer or collective movement (Li et al. 2017; Iacopini et al. 2024; Ramos-Fernandez et al. 2026) have exemplified how explicitly considering higher-order interactions can give a more complete understanding of emergent group-level outcomes and help to represent complex data with less information loss (Silk 2023). Fission-fusion dynamics, for example, where individuals in a group exhibit variable group sizes and compositions, potentially driven by collective foraging (Ramos-Fernandez et al. 2006, 2026), naturally exemplify the applicability of higher order networks. The second commonality is that information sharing typically requires spatial proximity. This second point suggests that the higher-order spatial distribution of the collective may intrinsically influence their effectiveness at collecting distributing foraging knowledge. The key pre-existing hypotheses relating higher-order spatial structure and information processing are the *recruitment centre hypothesis* (Evans et al. 2016) and *information centre hypothesis* (Evans et al. 2016; Richner and Heeb 1996), which both propose that higher-order spatial congregations, typically including all group members in a shared central area, allow for effective collective information processing, in turn allowing the group to be more adaptive to ecological and environmental variation. However, to the best of our knowledge, it is currently an open question which spatial structures are exactly *optimal* for collective information processing.

One important aspect of natural collectives is within-group heterogeneity in group foraging abilities, which we may expect particularly in an ecological context (although see Kaminka and Douchan (2025) to see the applicability in swarm robotics). In animal systems, there may be variation in individual abilities to move (Nathan et al. 2008), or willingness to gather or provide information (Jeanson and Weidenmüller 2013), which translate to variation in abilities to forage or gather other kinds of information. Many factors may influence this variation, such as differences in metabolic rate (Biro and Stamps 2010), cognitive ability (Manattini et al. 2024; Kashetsky et al. 2021), social position (Marshall et al. 2015; Stephens et al. 2007; Marshall et al. 2012), social affinities (Nathan et al. 2008), personality (Aliperti et al. 2021), age (Martins et al. 2024; Froy et al. 2015) or sex (Reyes-González et al. 2021; Ruckstuhl and Neuhaus 2006). If the group is to organise to maximise their collective potential for information sharing, then these heterogeneities should be accounted for. Such an optimal organisation might be expected when the individual benefits of information sharing outweigh the costs (Parker and Sutherland 1986; Robinson 1986; Romano et al. 2022), with the importance of this (specifically collective) optimisation enhanced when multilevel selection acts on group-level benefits and we expect kin selection (Fisher et al. 2017; Philson et al. 2024). For example, under the optimal strategy for the group as a whole, a forager with greater foraging abilities than the rest of the group may share more information than others, possibly leading to the costs of this behaviour outweighing the benefits for said individual. In such a scenario, we may not expect the (group-level) optimal structure to evolve due to unequal distribution of individual-level costs, and the forager with greater foraging abilities may even seek out a different group, or opt for solitary living (Brown et al. 1990; Robinson 1986). It is therefore important to examine how variation in foraging ability may translate to differences in both the spatial composition which is optimal for information sharing at the group-level, and in the individual-level costs to this spatial organisation.

Another important aspect of spatial composition is *group size*, or the number of individuals within the collective system. In robot swarms, the number of robots is determined by the type and size of the task and by project budgets, with the costs of single robots varying dramatically between applications (Schroeder et al. 2019). Theoretical studies suggest that there is an optimal swarm quantity because, while increasing the number of robots initially improves task performance, there can be diminishing marginal returns due to possible collisions and task interference (Lerman and Galstyan 2002). In animal systems, there is considerable variation in group size both between and within species (Brown et al. 1990; Chapman et al. 1995), and this variation is a key aspect in the balance of the costs and benefits to group-living and social behaviour as a whole (Silk 2007; Brown et al. 1990; Markham et al. 2015; Chapman and Valenta 2015; Wittenberger and Hunt 1985). In resource-constrained environments, larger groups may be more prone to within-group competition (Lee and Kim 2018; Seiler and Robbins 2020; Alexander 1974) and be associated to reduced individual reproductive rates (Borries et al. 2008), but may be more effective at collecting and sharing foraging information (Gibbs et al. 1987; Cantor et al. 2020; Giraldeau 1984; King and Cowlishaw 2007). Indeed empirical studies have found a positive relationship between resource availability and group size (Seiler and Robbins 2020; Gibbs et al. 1987), which may be related to the decreased influence of resource competition in more abundant environments. In sum, group-size is a vital component in a collective’s capacity to process and transfer information, and larger groups may not always be more effective at collecting and distributing information. It is therefore important to consider both how it may influence the properties of the optimal group spatial structure and how operating under optimal regimes may select for a particular group size.

In Ramos-Fernandez et al. (2026), we propose that the spatial structure of the group that is optimal for sharing knowledge may represent a balance between the collective benefit of further exploration by individuals and their opportunities for sharing with others. Specifically, this balance implies that the intersection between a set of individual areas should be large enough for them to coincide and exchange knowledge but not so large that they do not have any unique areas known to share information about. As a component of this model we quantified the amount of knowledge sharing for a certain class of spatial structures and used an optimisation approach to estimate the optimal structure (to be used as a baseline for comparison with the empirically observed spatial structure of a group of Geoffroy’s spider monkeys (*Ateles geoffroyi*), a classical example of species with a high degree of fission fusion dynamics (Ramos-Fernandez et al. 2006, 2026)). However, the optimality framework assumed that all individuals were equal in their foraging ability and that there was no constraint on resource availability, despite the importance of these factors in real systems. Considering these additional factors, and how they influence spatial structures under an optimal regime, could improve our general knowledge regarding the drivers of variation in natural spatial structures of foraging groups, for which we lack general theory.

In this study we generalise and formalise the optimisation component within the model of Ramos-Fernandez et al. (2026) to determine the higher-order spatial structure of a group of independent foragers which optimises the group-level transfer of knowledge when individuals vary in their information gathering ability and are placed in resource-constrained environments. The optimal structure is determined as the optimal balance between the opportunities for individuals to coincide and the relative uniqueness of their knowledge, represented here as the solution to a constrained integer-valued optimisation problem. Our model utilises an abstraction of the information environment into an arbitrary collection of foraging sites, allowing us to draw broader (topological) conclusions which are independent of any specific geometry. Each variable quantifies the number of ‘resource’ points shared uniquely within the core area overlap of any possible subset of individuals. By exploring the parameter space of this model we consider: 1) the impact of variation in foraging abilities upon the optimal spatial composition of the group; 2) the impact of resource sparsity upon this structure; and 3) how these results vary with group-size, whenever computationally feasible. This paper is structured as follows: in Section 2 we describe our model, the optimisation procedure and our methods for exploring parameter space. In Sections 3, 4 and 5 we explore model outcomes in three ‘scenarios’, each varying model parameters in a different way and representing increasing extents of within-group heterogeneity. In Section 6 we discuss our key model outcomes in the context of behavioural and evolutionary ecology, highlighting potential applications towards more general systems of collective intelligence. In an ecological context, our model reveals the significant role of individual foraging abilities in the spatial structure of groups of all sizes when organising solely for optimal knowledge transfer, and shows how the importance of differences in foraging abilities might covary with resource availability. While our model, as with many artificial collective systems, is inspired by animal social behaviour, it has broad applications beyond ecology, for example in the design of swarm robotic systems.

## Methods

We represent the higher-order spatial structure of a collective system which maximises their group-level information transfer as the solution to a constrained mixed integer quadratic programming (MIQP) problem. Particularly, the optimal spatial structure is the maximiser of some information transfer function, *T*, subject to some constraints reflecting resource abundance and individual capacities for sharing. This solution describes how knowledge of foraging sites is distributed between subsets of individuals without reference to the spatial distribution of the points of knowledge themselves. The solution therefore provides a *topological* description of the optimal group spatial structure. We develop quantitative measures of the optimal spatial structure, in order to conceptualise and contrast different higher-order structures. These allow us to study how individual abilities to forage, represented through knowledge of foraging points, and the amount of resources of the environment may drive variation in optimal spatial structures. We consider this variation through three ‘scenarios’, with sequentially increasing within-group heterogeneity in foraging ability.

### Problem Variables and Parameters

Let *n* be the number of individuals within the environment/group. The foraging environment is represented as a collection of $F\in \mathbb {N}$ many points (taking the convention $0\notin \mathbb {N}$), each representing a potential foraging site. Each individual *i* has a knowledge capacity of $N_i\in \mathbb {N}$ many points for $i\in [n] := \{1,\dots ,n\}$. We assume that individuals utilise their knowledge capacity as far as their environment will allow, so an individual with capacity $N_i$ will have an *effective* knowledge of $N_i'\in \mathbb {N}$ many points, where

1 $$\begin{aligned} N_i' = \min \{N_i, F\}. \end{aligned}$$

**Fig. 1:**
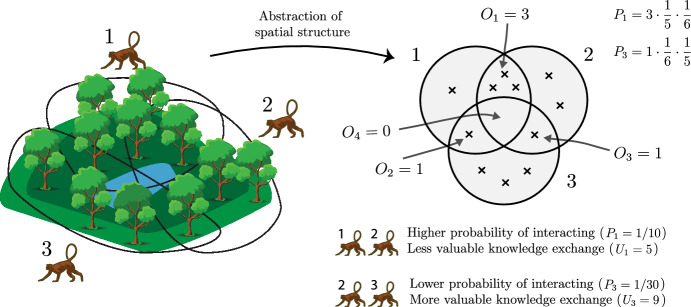
Conceptualisation of the foraging environment and problem variables. The knowledge points represent, in this case, the trees in which food may be available. In this example, individuals 1 and 2 have relatively less unique knowledge, but higher probability of interaction, in contrast to individuals 2 and 3 (color figure online)

Each point can be known uniquely by either; 1) zero individuals, 2) a single individual, or 3) a *subgroup* of individuals (here exclusively referring to groups of at least 2 individuals). By this definition, the set of all possible subgroups, $\mathcal {X}$, is obtained as the power set of [*n*] minus singletons and the empty set, taking cardinality $|\mathcal {X}| = \mathcal {N}(n) = 2^n-n-1$ which is the dimensionality of our optimisation problem (henceforth being denoted as $\mathcal {N}$ for notational simplicity). We order this set by first arranging the elements in ascending cardinality (so interactions between fewer individuals come before interactions between more individuals) and then arranging elements of the same cardinality lexicographically. Denote the bijection corresponding to this ordering by $I:\mathcal {X}\rightarrow [\mathcal {N}]$. The variables of the optimisation problem are $\boldsymbol{O}=(O_i)\in \mathbb {N}^{\mathcal {N}}$, where $O_i$ gives the number of points uniquely known by the subgroup with index *i* under enumeration *I*. These variables are exemplified in Figure 1.

Note that $\boldsymbol{O}$ fully describes the higher-order spatial structure, since the number of cells known by only a single individual *i* can be determined by their own effective foraging knowledge, $N_i'$, minus their number of shared points within subgroups, and the number of points known by zero individuals can then similarly be determined from *F*. We remark that, together with the bijection *I*, the vector $\boldsymbol{O}$ fully defines a *weighted hypergraph* (a higher-dimensional generalisation of a weighted graph or network which allows edges between any number of individuals; Berge (1989); Silk et al. (2022)). The corresponding hypergraph to $\boldsymbol{O}$ has nodes [*n*], hyperedges between *two or more* individuals given by $\mathcal {X}$ with corresponding edge weight $O_i$ for $i\in [\mathcal {N}]$ and hyperedges *for single individuals* given by [*n*] with corresponding edge weight being the individuals own unique knowledge.

### Objective Function

We assume that individuals move between their points of knowledge uniformly, so the probability that the *i*-th individual occupies a particular point at a given time is

$$\begin{aligned} p_i = \frac{1}{N_i'}. \end{aligned}$$

Then, assuming that individuals move independently, the probability of the *c*-th subgroup (under enumeration *I*) coinciding at the same foraging point under the spatial structure $\boldsymbol{O}$ is given as

$$\begin{aligned} P_c\left( \boldsymbol{O}\right) = \left( \prod _{k\in I^{-1}(c)}p_k\right) \left( \sum _{a\in S(c)} O_a\right) = \frac{\sum _{a\in S(c)} O_a}{\prod _ {k\in I^{-1}(c)}N_k'} \end{aligned}$$

where

$$\begin{aligned} S(c) = \left\{ i\in \left[ \mathcal {N} \right] : I^{-1}(c)\subseteq I^{-1}(i)\right\} \end{aligned}$$

gives the indices of subsets which include the *c*-th subgroup for all $c\in \left[ \mathcal {N}\right]$. Under this construction, any subgroup of individuals can interact in both the areas that they uniquely have knowledge of *and* in the areas known by them and additional individuals. We then assume that when a subgroup meets they share their *collective information*. This is defined as the number of points known by at least one individual in the subgroup, but not by all individuals. For the *c*-th subgroup, this is given by

$$\begin{aligned} U_c(\boldsymbol{O}) = \underbrace{\sum _{k\in I^{-1}(c)}N_k'}_{\text {Total knowledge in }I^{-1}(c)} - \underbrace{f(c)\sum _{a\in S(c)}O_a}_{\text {Points known by \textit{all} of }I^{-1}(c)}-\underbrace{\sum _{a\in B(c)}(f(a)-1)O_a}_{\text {Over-counted points}} \end{aligned}$$

where

$$\begin{aligned} B(c) = \left\{ i\in \left[ \mathcal {N} \right] : I^{-1}(i) \subset I^{-1}(c)\right\} \end{aligned}$$

is the set of indices of all the smaller subgroups of individuals contained in the *c*-th subgroup of individuals under the enumeration *I*, and $f:[\mathcal {N}]\rightarrow [n]$ is the size of the *c*-th subgroup, $f(c)=|I^{-1}(c)|$.

We then assume that the rate of information transfer when the subgroup coincides is directly proportional to this uniquely known area. The net information transfer across the entire group of *n* individuals is then given by $T:\mathbb {R}^{\mathcal {N}(n)} \rightarrow \mathbb {R}$

$$\begin{aligned} T(\boldsymbol{O})&= \sum _{c=1}^\mathcal {N} P_c(\boldsymbol{O})U_c({\boldsymbol{O}}) \\&= \sum _{c=1}^\mathcal {N} \left( \frac{\sum _{a\in S(c)} O_a}{\prod _ {k\in I^{-1}(c)}N_k'}\right) \left( \sum _{k\in I^{-1}(c)}N_k' - f(c)\sum _{a\in S(c)}O_a-\sum _{a\in B(c)}(f(c)-1)O_a \right) . \end{aligned}$$

Observing that this is a quadratic form, we can write

$$\begin{aligned} T(\boldsymbol{O}) = \boldsymbol{l}^t\boldsymbol{O}+\frac{1}{2}\boldsymbol{O}^tM\boldsymbol{O} \end{aligned}$$

for some $\boldsymbol{l}\in \mathbb {R}^{\mathcal {N}}$ and $M\in \mathbb {R}^{\mathcal {N}\times \mathcal {N}}$. In Section S1.1 we explicitly derive the coefficients of the vector $\boldsymbol{l}$ and matrix *M*.

### Constraints

Our problem constraints are determined by the following; 1) individuals must share a non-negative number of points within subgroups, 2) individuals cannot share more points than they have knowledge of, and 3) the group as a whole cannot have knowledge of more than *F*-many foraging points. Respectively, this yields the constraints

2 $$\begin{aligned}&\boldsymbol{O}\ge \boldsymbol{0}, \nonumber \\&\sum _{i\in \tilde{S}(k)}O_i\le N_k', \text { for all } k\in [n], \end{aligned}$$

3 $$\begin{aligned}&\sum _{i=1}^nN_i' - \sum _{c=1}^\mathcal {N}(f(c)-1)O_c \le F, \end{aligned}$$

where $\tilde{S}(k)$ is the set of indices of subgroups which contain the *k*-th individual, given explicitly by

4 $$\begin{aligned} \tilde{S}(k) = \left\{ i\in [\mathcal {N}]: \{k\}\subset I^{-1}(i)\right\} . \end{aligned}$$

Equation (3) sums over each individual’s effective knowledge and then accounts for over-counted points due to knowledge sharing under the spatial structure $\boldsymbol{O}$.

Whenever $F\ge \sum _{i=1}^nN_i'$ the constraint (3) is automatically satisfied by the non-negativity of $\boldsymbol{O}$. We refer to this case as ‘resource-abundant’, and the case where $F<\sum _{i=1}^nN_i'$ as ‘resource-constrained’. The constraints (2) and (3) are both linear, so can be collectively written in the form $G\boldsymbol{O} \le h$, where $G\in \mathbb {R}^{(n+1)\times \mathcal {N}}$ and $h\in \mathbb {R}^{n+1}$. The specific entries of *G* and *h* are given in Section S1.2.

### Optimisation Procedure

The maximisation problem of the objective function, *T*, defined in Subsection 2.2 subject to the constraints defined in Subsection 2.3, written as

5 $$\begin{aligned} \max _{\boldsymbol{O}}&\; \quad T(\boldsymbol{O}) = \boldsymbol{l}^t\boldsymbol{O}+\frac{1}{2}\boldsymbol{O}^tM\boldsymbol{O}\nonumber \\ \text {s.t}&\; \begin{aligned} \quad G\boldsymbol{O}&\le h \\ \boldsymbol{O}&\ge \boldsymbol{0} \\ \boldsymbol{O}&\in \mathbb {Z}^{\mathcal {N}(n)} \end{aligned} \end{aligned}$$

is feasible whenever $F\ge 0$ (see Section S1.3). We solve this MIQP using a suite of global optimisation solvers: *Gurobi* (Gurobi Optimization 2024), *IBM ILOG CPLEX*, and *FICO Xpress*, all obtained through their free academic licences and implemented in Python. Each of these solvers utilise a branch-and-cut method for solving MIQPs. With each combination of parameters (i.e. each value of *F*, *n* and $\boldsymbol{N}=(N_i)_{i\in [n]}$) considered in this study, the corresponding optimisation problem is solved independently with each solver, and the ‘best’ solution (that which provides the largest maximal value of *T*), $\boldsymbol{O}^*$, is returned.

**Table 1:** Description of the quantitative measures of the higher-order spatial structure used in this study, alongside their respective formulae and range of values taken. Here, $\boldsymbol{O}=\left( O_c\right) \in \mathbb {R}^\mathcal {N}$ is the description of the higher-order structure, as defined in Section 2.1, $l\in \mathbb {R}^{\mathcal {N}}$ and $M\in \mathbb {R}^{\mathcal {N}\times \mathcal {N}}$ are the parameters of the objective function defined in Section 2.2 and derived explicitly in Section S1.1, ${\boldsymbol{1}}_i:[n]\rightarrow \{0, 1\}$ is the indicator function (with ${\boldsymbol{1}}_i(x)=1$ if $x=i$ and ${\boldsymbol{1}}_i(x)=0$ otherwise), $f(c)=| I^{-1}(c)|$ is the size of the *c*-th subgroup for $c\in [\mathcal {N}]$, $N_k'$ is the effective knowledge of the *k*-th individual defined in Equation (1), and $\tilde{S}(k)$ is the set of indices of subgroups containing the *k*-th individual, given in Equation (4). Measures are always evaluated at the optimal spatial structure, $\boldsymbol{O}^*$, obtained as the solution to the MIQP (5), solved according to the procedure outlined in Section 2.4

**Measure**	**Description**	**Formula**	**Range**
Information Transfer, $T\left( \boldsymbol{O}\right)$	Value of the objective function, describing the efficiency of the higher-order spatial structure for collecting and distributing information, according to the construction and assumptions outlined in Section 2.2.	$\boldsymbol{l}^t\boldsymbol{O}+\frac{1}{2}\boldsymbol{O}^tM\boldsymbol{O}$	$[0, \infty )$
Relative *i*-th Order Overlap, $i\ge 2$, $w_n^{(i)}(\boldsymbol{O})$	Proportion of foraging points known uniquely by subgroups of size *i* in a group of size *n* (relative to the number of points known by at least one individual in the group).	$\frac{\sum _{c=1}^\mathcal {N}{\boldsymbol{1}}_i(f(c))O_c}{ \sum _{j=1}^nN_j' - \sum _{c=1}^\mathcal {N}(f(c)-1)O_c}$	[0, 1]
Relative Uniquely-Known Space, $w_n^{(1)}\left( \boldsymbol{O}\right)$	The proportion of points known by *exactly one* individual in a group of size *n* (relative to the number of points known by at least one individual in the group).	$w^{(1)}_n(\boldsymbol{O}) = 1 - \sum _{i=2}^nw_n^{(i)}(\boldsymbol{O}).$	[0, 1]
Expected Crowding, $K(\boldsymbol{O})$	Expected number of individuals with knowledge of a given point. Higher values therefore correspond to a higher amount of crowding around foraging points, which may reflect increased competition, for example.	$\sum _{i=1}^n i w_n^{(i)}(\boldsymbol{O})$	[0, *n*]
Proportion of Shared Points for Individual *k*, $C_k(\boldsymbol{O})$	Describes, for individual *k*, the proportion of its own known foraging points which are known by least one additional individual. Higher values correspond to a higher proportion of points shared with others, and therefore a lower amount of unique foraging knowledge.	$\frac{\sum _{i\in \tilde{S}(k)}O_i}{N_k'}$	[0, 1]

### Measures of the Spatial Structure

To quantify the properties of the optimal spatial structure, we develop descriptive measures of the higher-order spatial structure, defined explicitly in Table 1. For $i=2,\dots , n$ we define the *relative* *i-th order overlap* of the spatial structure as $w_n^{(i)}$ for $i\in [n]$, which describes the proportion of foraging points known uniquely by subgroups of size *i* (relative to the number of points known by at least one individual in the group). Higher values of $w_n^{(i)}$ therefore indicate a greater extent of space sharing *specifically* between *i*-many individuals in the spatial structure $\boldsymbol{O}$.

We then define the *expected crowding*, $K(\boldsymbol{O})$, in terms of the proportions $w_n^{(i)}$ (see Table 1). This gives the expected number of individuals which have knowledge of a given foraging point. Lower values of $K(\boldsymbol{O})$ indicate each point is known by fewer individuals on average, suggesting that there might be less crowding of individuals around foraging points, potentially implying lower individual-level costs, particularly in the context of food sharing, as resources could be shared with fewer individuals and therefore subject to weakened competition. Meaning, resources could be considered as ‘higher value’. This metric can take values between 1 (when no space is shared by any individuals, so $w_n^{(1)} = 1$ and $w_n^{(i)}=0$ for $i=2,\dots ,n$) and *n* (when all space is shared by all individuals, so $w_n^{(n)} = 1$ and $w_n^{(i)}=0$ for $i=1,\dots ,n-1$).

To quantify these potential costs of sharing at an individual-level, we define the *proportion of shared points for individual* *k*, $C_k(\boldsymbol{O})$ for $k\in [n]$, as the proportion of points that the *k*-th individual shares with *any other* individual (see Table 1)^1^. Higher values correspond to a higher proportion of points shared with others and therefore a lower proportion of uniquely known points, which may represent some individual-level cost (Lee et al. 2016) occurred with the spatial structure $\boldsymbol{O}$ (describing the amount of competition or resource sharing that individual *k* may observe in the spatial structure *O*). Note that this potential cost is specific to a particular individual, whereas *K* works at the level of the group.

We additionally report the value of the objective function at the optima, $T(\boldsymbol{O}^*)$, but note that the value of *T* is not designed to be continuous between groups of different sizes as one member disappears. Meaning the value of *T* defined over $\mathbb {R}^{\mathcal {N}(n)}$ as $N_i$ approaches 0 does not converge to the value of *T* on $\mathbb {R}^{\mathcal {N}(n-1)}$ for the remaining $n-1$ members. However, all other measures introduced here can be meaningfully compared because the spatial structure corresponding to the solution $\boldsymbol{O}^*$ is consistent in this limit.

### Scenarios

We consider how the solution to the MIQP (5) varies with individual abilities, $\boldsymbol{N}$, and resource abundance, *F*, through different scenarios, described in Table 2. Each scenario considers sequentially greater heterogeneity in $\boldsymbol{N}$ than the last. The first scenario (*Homogeneous Knowledge*) assumes a homogeneous population, where all individuals have the same knowledge gathering abilities, functioning effectively as a baseline model or a scenario particularly relevant for artificial systems like robot swarms. The next scenario (*Distinct Forager*) examines how optimal spatial structures may change when a single individual varies in their foraging abilities, representing an initial examination into how heterogeneity may influence optimal structures. This scenario is of strong ecological relevance, allowing us to examine if/how socially or physiologically unique individuals might take unique positions within the social structure. These unique individuals may also represent some mutation of a phenotype related to foraging behaviour. The final scenario (*Heterogeneous Knowledge*) allows us to examine heterogeneity explicitly at the level of the entire group, where we fix the mean foraging ability but increase the variance in these abilities, moving from a homogeneous group to one with significantly overdispersed foraging abilities. We choose a unimodal distribution with low skewness, a *truncated normal distribution*, to generate each $N_i>0$ independently. Low skewness helps to ensure our results are linked to general variation in foraging abilities rather than variation in a particular direction. This low skewness will be maintained for a range of $\sigma$ values, but for sufficiently large $\sigma$ positive skewness is inevitable given the condition $N_i>0$.

Our explicit consideration of these cases distinctly allows us to extract the mechanistic relationship between heterogeneity and optimal spatial structure for collective foragers placed in environments with varied resource availability. For each of these scenarios, we consider the role of both group size *n* (where computationally feasible due to exponential growth in problem dimensionality, $\mathcal {N}$, with *n*) and resource abundance *F* upon the optimal spatial structure, differentiating between the resource-abundant and resource-constrained scenarios.

**Table 2:** Summary of the different foraging scenarios used in the study, detailing the individual knowledge structures and the parameters varied in the simulations. Consistently throughout we use $N=10^4$. When parameters are varied, we calculate the measures shown in Table 1 for each parameter combination to evaluate how the optimal spatial structure changes. Due to computational constraints, we only consider the $n=5$ case in the heterogeneous knowledge scenario, so we conduct 500 Monte Carlo simulations for each scenario in to counter the low sampling rate from the underlying distribution of $N_i$. The number of points sampled within each shown interval is given in the relevant figure captions

**Scenario**	**Section**	**Individual abilities** $\boldsymbol{N}=\left( N_i\right) _{i\in [n]}$	**Parameters varied**
Homogeneous knowledge	3	$N_i=N$ for some $N\in \mathbb {N}$.	$F\in [0.01N , 4N]$, $n\in \{3,4,5,6,7\}$
Distinct forager	4	$N_i=N\in \mathbb {N}$, except for one individual with ability $N_1=DN$ for $D\in \mathbb {R}^{>0}$.	$D\in [0.01, 6.00]$, $F\in [0.01N, 4N]$, $n\in \{3,4,5,6,7\}$
Heterogeneous knowledge	5	$N_i$ drawn independently according to a normal distribution truncated and translated to have mean $N\in \mathbb {N}$, standard deviation $\sigma \ge 0$ while ensuring $N_i\ge 0.5$ (for full details, see Section S1.4). Each $N_i$ is then rounded so that $N_i\in \mathbb {N}$ for each $i\in [n]$. We run Monte Carlo simulation with 500 simulations for each value of $\sigma$.	$\sigma \in [0, N]$, $F\in [0.01 N, 4N]$, $n=5$

## Homogeneous Knowledge Scenario

This scenario represents the simplest group composition, where all individuals have exactly the same ability to forage ($N_i=N\in \mathbb {N}$ for all $i\in [n]$). We discuss the resource-abundant case as a baseline for comparison with the resource-constrained case.

### Resource-Abundant Environment

In this case, our model is equivalent to that of Ramos-Fernandez et al. (2026), with the additional formalisation of integer-valued variables and the inclusion of our additional descriptive measures. The solution to the information sharing optimisation problem (5) is

6 $$\begin{aligned} \boldsymbol{O}^* = \left( 0,\dots , 0, \frac{N}{2}\right) \end{aligned}$$

when *N* is even, and a similar solution is returned when *N* is odd, either rounding up or down the final entry^2^. In Section S2.1 we directly prove that $\boldsymbol{O}^*$ is the *global* optima of *T* in this homogeneous, resource-abundant case. This corresponds to an exact balance between exploration and sharing; each individual shares half of their space with *all* other individuals in a centrally shared area, and the other half is not shared with any other individual, with no lower-order space sharing occurring. This aligns with the information centre hypothesis in ecology, which suggests that individuals share a common area as an effective means to collect and distribute information across the group (Evans et al. 2016). Here, the spatial structure described by $\boldsymbol{O}^*$ has the characteristics:

$$\begin{aligned} w_{n}^{(1)} = \frac{n}{n+1}, \hspace{10pt}w_n^{(n)} = \frac{1}{n+1}, \hspace{10pt} w_n^{(i)} = 0 \text { for } i = 2, \dots , n-1, \end{aligned}$$

$$\begin{aligned} K = \frac{2n}{n+1}, \hspace{10pt}C_k = \frac{1}{2} \text { for all } k\in [n] \end{aligned}$$

with the corresponding information transfer

$$\begin{aligned} T(\boldsymbol{O}^*) = \frac{N^2}{2}\sum _{k=2}^n {{n}\atopwithdelims (){k}}kN^{-k} = \frac{n(n-1)}{4} + \mathcal {O}(N^{-1}) \end{aligned}$$

as derived in Section S2.2. Therefore, as the group size *n* increases, the group knowledge transfer increases approximately quadratically (asymptotically with large *N*), while each individual maintains the same proportion of uniquely known points ($C_k$ is independent to *n*) and the group shares a lower proportion of their points ($w_n^{(n)}$ is decreasing in *n*). In larger groups, each point is known by more individuals on average (*K* is increasing in *n*) due to the larger number of individuals in the shared central area. These changes in $w_n^{(n)}$ and *K* become smaller in magnitude as *n* increases due to the asymptotic nature of these quantities, and so the effect of increasing group size is diminishing.

### Resource-Constrained Environment

**Fig. 2:**
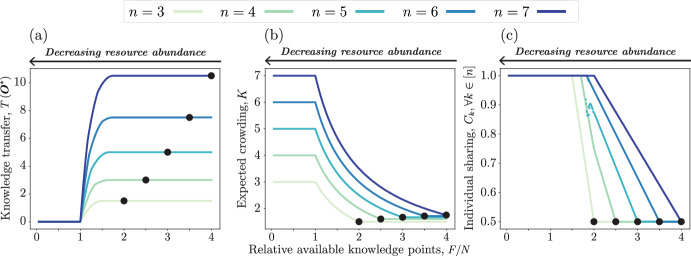
Quantitative descriptions of the optimal spatial structure in the homogeneous scenario ($N_i=N=10^4$ for all $i\in [n]$) with variation in resource abundance, $F\in [0.05N, 4N]$, with values plotted relative to *N*. Specific values of *F* are obtained from 1000 uniformly spaced points in this interval rounded upwards so $F\in \mathbb {N}$. Shown are: (a) the value of the objective function at the optimal solution, $T(\boldsymbol{O}^*)$, (b) the expected number of individuals with knowledge of each point under the optimal structure, $K(\boldsymbol{O}^*)$, and (c) the proportion of each individual’s *N*-many points which are also known by other individuals, $C_k$ (which is fixed across $k\in [n]$ by problem symmetry). Darker colours correspond to higher group sizes, varying sequentially from $n=3$ to $n=7$. Black dots mark the critical relative foraging abundance, $F^*/N=(n+1)/2$, where the resource-abundant optimal structure changes feasibility, for the corresponding group size *n*. The disturbance in the graph in (c) for $n=5$ is suspected to be related to the non-concavity of the objective function resulting in multiple global optima (color figure online)

The optimal structure from the unconstrained foraging environment makes use of $N(n+1)/2$ many points (*N*/2 from the central shared space, and *nN*/2 from the remaining uniquely known points by all individuals), such that this solution remains feasible and optimal as long as $F\ge F^* =N(n+1)/2$. This critical value, $F^*$ is increasing with *n*, such that in larger groups the resource-abundant optimal structure remains feasible only with increasingly large values of *F*. As *F* decreases below the critical value $F^*$, there is initially negligible decrease in $T\left( \boldsymbol{O}^*\right)$ (Figure 2a), such that groups are robust to decreases in the resource abundance of certain sizes. With this decreasing abundance there is an increase in *K* (each point is known by more individuals due to the lower abundance of points) until $F=N$ (where *K* reaches its maximum value of $K=n$; Figure 2b). We also find that $C_k$ increases as *F* decreases, so that in more harshly constrained environments individuals must share a higher proportion of their uniquely known points in order to maximise the group-level benefit (Figure 2c). This additional sharing in the optimal structure appears rapidly with decreasing *F*, and individuals lose all of their unique knowledge ($C_k = 1$) before reaching the trivial structure observed at $F=N$ (compare Figures 2b and 2c). The change in the objective value *T* when decreasing *F* is small to begin with, even when $C_k=1$, but plummets to 0 when *F* is close to *N* and the completely-shared structure is forced (Figure 2a). In this completely-shared structure, both *K* and $C_k$ are maximal, but *T* is minimal because no subgroup of individuals holds any unique information. This suggests that reorganisation to maintain group-level information transfer in adaptation to resource constraints is effective but that individual-level costs, such as decreased value of knowledge points, are incurred by forming this structure. All of these behaviours are qualitatively similar across tested group sizes (Figure 2).

**Fig. 3:**
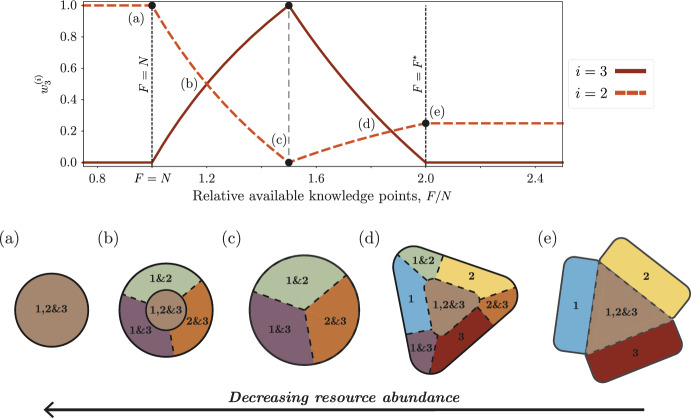
Variation in the proportion of higher-order overlaps with the foraging point abundance relative to knowledge, $F/N\in [0.75, 2.50]$ in the homogeneous scenario ($N_i=N=10^4$ for all $i\in [n]$) for group size $n=3$. Specific values of *F* are obtained from 1000 uniformly spaced points in this interval rounded upwards. Top: (red solid line) $w_3^{(2)}$, the proportion of dyadic point sharing and (orange dashed line) $w_3^{(3)}$, the proportion of points uniquely shared between all three individuals. Bottom: Conceptual representations of the optimal spatial structures (using areas instead of a collection of points) corresponding to points along these plots are marked (a)-(e), with (e) representing the optimal spatial structure in the resource-abundant scenario, namely $\boldsymbol{O}^* = \left( 0, 0, 0, N/2\right)$ (color figure online)

**Fig. 4:**
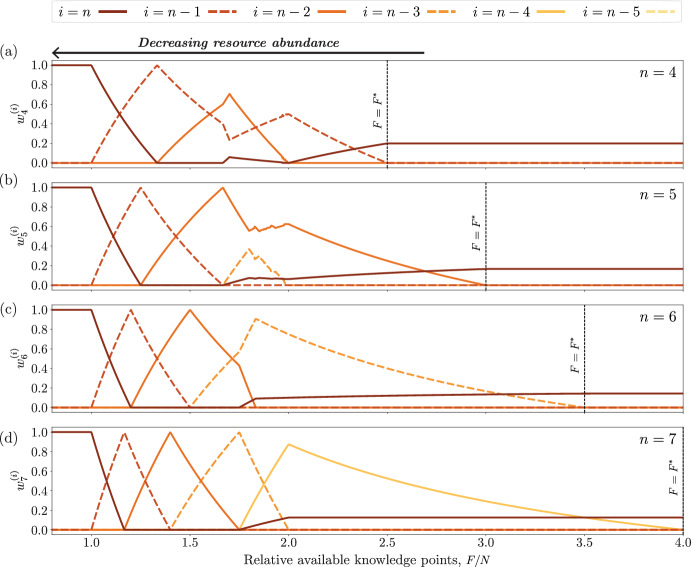
Variation in the order of spatial overlap, represented by $w_n^{(i)}$, in the homogeneous scenario ($N_i=N=10^4$ for all $i\in [n]$) with varying *relative* resource abundance $F/N\in [0.75, 4.00]$. Specific values of *F* are obtained from 1000 uniformly spaced points in this interval rounded upwards. Group sizes are: (a) $n=4$, (b) $n=5$, (c) $n=6$, and (d) $n=7$. Darker colours correspond to orders of space sharing closer to *n*. The sequential dashed and solid lines are for plot clarity only. The noisy region in the graph in (c) for $n=5$ is suspected to be related to the non-concavity of the objective function resulting in multiple global optima (color figure online)

When initially decreasing *F* from $F^*$ in the case of $n=3$, as visualised in Figure 3, there is initially a gradual switch between order-3 space sharing ($w_3^{(3)}$) and order-2 $w_3^{(2)}$ space sharing, until the structure consists eventually entirely of dyadic space sharing ($w_3^{(2)}=1$). After this point, no lower-order interactions can be engaged in, so there is forced sharing between all group members which increases until a trivial structure is reached. This observation generalises to larger group sizes as follows. When initially decreasing *F* from $F^*$ the proportion of maximal-order space sharing, $w_n^{(n)}$, decreases towards 0 and the proportion of some lower order spatial overlap, ($w_n^{(i)}$ for some $i<n$) increases towards some peak value (Figures 3 and 4). When *F* decreases beyond this peak in $w_n^{(i)}$, a similar switch occurs between the values of $w_n^{(i)}$ and $w_n^{(i+1)}$; the one-higher order spatial sharing takes place in the optimal structure instead of the lower-order. As the proportion of the lower-order space sharing reaches zero, the higher order reaches its peak, and the switch occurs again with a further higher-order sharing. This pattern continues until the peak in $w_n^{(n-1)}$, after which the proportion of centrally shared space $w_n^{(n)}$ increases from 0 to 1, indicating the forced sharing of space which occurs as *F* approaches *N*. In short, particularly high or low values of *F* can promote higher-order sharing, while intermediate values will typically promote lower-order sharing. We describe this as a *pattern of interaction order selection*.

## Distinct Forager Scenario

In this scenario we considered variation in the foraging ability of just a single individual (called the *distinct forager*), taking $\boldsymbol{N}=(DN, N, \dots , N)$ for some $D>0$. When $D<1$, the distinct forager has a lower ability (the *one-worse* scenario), and when $D>1$ they have a higher ability (the *one-better* scenario). We consider the impact of variation in *D* upon the optimal spatial structure of the group, and how this interacts with variation in resource availability *F*.

**Fig. 5:**
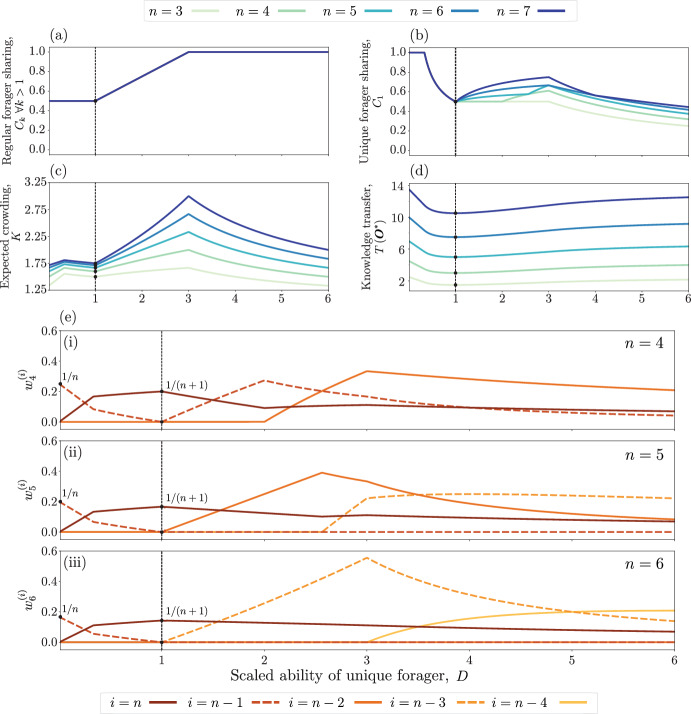
Results from the (resource-abundant) distinct forager scenario, where $\boldsymbol{N}=(DN, N, \dots , N)$ for $D\in [0.01, 6.00]$. Specific values of *D* are obtained from 1000 uniformly spaced points in this interval rounded upwards. Shown are: (a) the proportion of regular individual’s *N*-many points also known by other individuals, $C_k$ (for $k>1$), (b) the proportion of the distinct foragers *DN*-many points also known by other individuals, $C_1$, (c) the expected number of individuals with knowledge of each point under the optimal structure, *K*, (d) the value of the objective function at the optimal solution, $T(\boldsymbol{O}^*)$, and (e) the relative orders of spatial overlap, $w_n^{(i)}$, for $i=2,\dots , n$ with group size (i) $n=4$, (ii) $n=5$, (iii) $n=6$. In (a)-(d), darker colours correspond to higher group size ($n=4,5,6$), while in (e) they correspond to orders of space sharing closer to *n*. We assume that $F>F^*$, so we neglect the foraging constraint for each value of *D* (color figure online)

### Resource-Abundant Environment

In the one-worse scenario, as *D* *decreases* from $D=1$ (which represents the homogeneous scenario; Section 3), regular individuals do not sacrifice any more of their uniquely known points, represented by $C_k$ being constant for $k\ge 2$ and $D\in (0, 1]$, shown in Figure 5a. However, the distinct forager shares more of their own unique area and eventually all of it ($C_1$ is increasing until around $D=1/3$, at which point it takes its maximal value of $C_1=1$; Figure 5b), suggesting that the optimal strategy (specifically at the group-level) is for the worse forager to be ‘supported’ in an altruistic manner. However, in natural systems, since this behaviour is highly non-reciprocal, based on foraging behaviour alone we may not expect this kind of structure unless selection at the level of the group mostly drives spatial structure (rather than selection at the individual-level). Variation in the unique area held by each individual is consistent between the tested group-sizes. As *D* approaches 0 in a group of size *n*, the optimal structure from a homogeneous group of size $n-1$ expectedly emerges. Particularly, $w_n^{(n-1)}$ approaches 1/*n* and all other values of $w_n^{(i)}$ (for $i>1$) approach 0 (see Figure 5e). As mentioned in Section 2.5 the objective function value does not approach that of the lower group size since *T* is not “continuous” between group sizes as a single individuals knowledge approaches 0.

As *D* *increases* from $D=1$ (now representing the one-better scenario) both the distinct forager and the regular foragers share a higher proportion of their space with others (each $C_k$ is increasing; Figures 5a and 5b), leading to an increase in the expected sharing *K* (Figure 5c). This indicates that the optimal strategy for the group is for the distinct forager to share more space with others rather than exploring more points which are novel to the group (relative to the homogeneous structure). The increased sharing continues until $D=3$, when regular individuals share all their space with the distinct forager ($C_k = 1$ for $k\ge 2$), maximising their probability of interaction. Ecologically, this increased sharing implies that distinct foragers may end up in more central socio-spatial positions. Consequently, these individuals may face unique social and epidemiological pressures, and the structure of the collective itself may alter contagion dynamics (e.g. super-spreader dynamics (Lloyd-Smith et al. 2005)). In the design of collective systems, this implies that superior (up-to the threshold of $D=3$) individuals should focus on increased interaction with other individuals rather than increase their own exploration. As *D* increases far beyond this threshold, the optimal strategy is for the distinct forager to engage in additional exploration (necessarily, as it cannot share more points with others), as shown by the decrease in $C_1$ after $D=3$ (Figure 5b). Beyond the threshold, the group-level information transfer plateaus (Figure 5d), implying no additional group-level gains from improving the ability of a superior agent. Therefore, in the design of collective systems (e.g. in the set up of robotic swarms), there is a limiting advantage to introducing new information gatherers which are particularly stronger than the rest of the group. Furthermore, this implies that distinct foragers in animal systems may take the most central socio-spatial position at the threshold of $D=3$, when the distinct forager engages in maximal sharing, and the corresponding variation in the social or epidemiological dynamics may be greatest for such foragers. While there is variation in the proportion of space shared by the distinct forager across the tested group sizes (with the forager sharing more of their uniquely known points in larger groups; $C_1$ is increasing with *n*), the qualitative shift in the dynamics of the optimal structure with *D* after $D=3$ is consistently observed (Figure 5b), suggesting that this threshold may be of further theoretical interest.

**Fig. 6:**
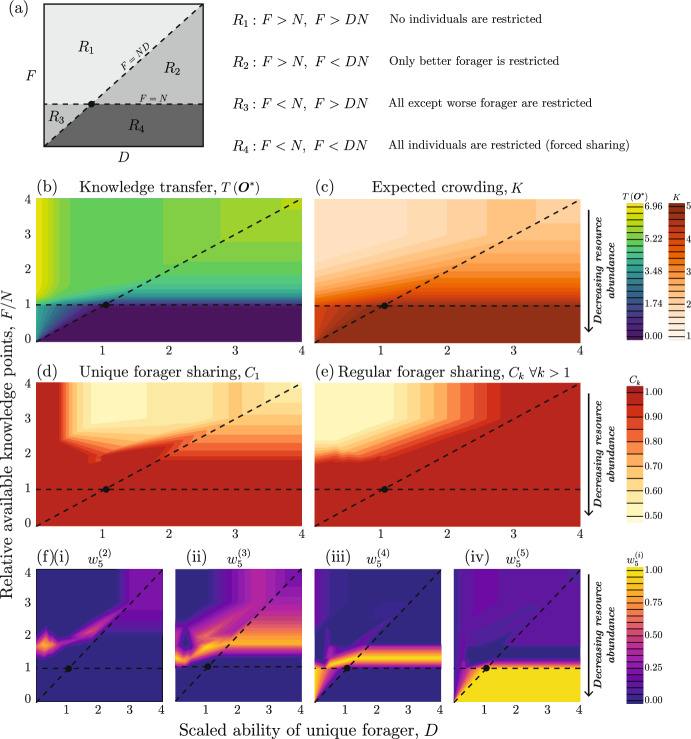
Results for the distinct forager scenario where $\boldsymbol{N}=(DN, N, \dots , N)$ with $D\in [0.01, 4]$, with number of foraging points (relative to the standard individual knowledge capacity *N*) varying between $F/N\in [0.05, 4]$, and for group size $n=5$. Specific values of *D* and *F* are obtained from 150 uniformly spaced points over their respective intervals, each rounded upwards. Shown are: (a) a schematic for the division of the (*D*, *F*) parameter space into four regions, as outlined in the main text, (b) the value of the objective function at the optimal solution, $T(\boldsymbol{O}^*)$, (c) the expected number of individuals with knowledge of each point under the optimal structure, *K*, (d) the proportion of distinct foragers (*DN*)-many points also known by other individuals, $C_1$, (e) the proportion of regular individual’s *N*-many points also known by other individuals, $C_k$ (for $k>1$, fixed by problem symmetry), and (f) the relative orders of spatial overlap $w_5^{(i)}$ for (i) $i=2$, (ii) $i=3$, (iii) $i=4$ and (iv) $i=5$. Dashed lines throughout show the division between regions $R_1$, $R_2$, $R_3$ and $R_4$. All regions fit within the resource-constrained region, defined by $F\le (n-1)N+DN$ (color figure online)

### Resource-Constrained Environment

We find that the optimal spatial structure is dependent upon the combination of the relative ability of the distinct forager, *D*, and the resource availability, *F*. In particular, variation in measures of the optimal structure across the (*D*, *F*) parameter space clearly separates the space into 4 regions, as defined in Figure 6a. These regions, denoted by $R_1$, $R_2$, $R_3$ and $R_4$, are defined by which individuals are ‘restricted’ (meaning whose effective knowledge is *F*, rather than their knowledge capacity $N_i$) by their environment for the given values of (*D*, *F*). Particularly, the distinct forager is restricted by their environment when $F<DN$, and the regular individuals are restricted when $F<N$. In Region $R_1$ we typically observe stronger dependencies upon the value of *D* (shown by the constant vertical regions which appear in many of the plots in Figure 6), whereas in region $R_2$ we *consistently* observe dependence only upon *F* (as shown by the constant horizontal regions in Figure 6). This is because the distinct forager is restricted to occupying *F* many points, and there is no impact of variation of their actual ability *D* within this region of parameter space. In region $R_3$, all recorded features vary monotonically with *D*, with the rate of variation being higher in more constrained systems (i.e. when *F* is lower). Finally, within Region $R_4$, all individuals in the group have foraging abilities greater than that of their environment, so are forced into sharing all of the *F*-many points available (due to the assumption that individuals utilise their foraging abilities as far as their environment will allow). In this case, where complete sharing is forced, there is no knowledge transfer due to the lack of any unique knowledge (each point is known by all individuals and the spatial structure is trivial, i.e. $U_c(\boldsymbol{O})=0$ $\forall c$, $w_n^{(n)}=1$ and $K=n$).

As in the homogeneous scenario (Section 3), we observe patterns of interaction order selection in how the optimal spatial structure varies with *F* (where increasing *F* led to the optimal social structure consisting of sequentially lower-order interactions). In particular, surrounding the border of region $R_4$ (where $w_n^{(n)}=1$) there is a region where $w_n^{(n-1)}$ peaks and $w_n^{(n)}$ falls, and surrounding this region there is another region where $w_n^{(n-2)}$ peaks and $w_n^{(n-1)}$ falls, and so on. This is exemplified in Figure 6f for the case of $n=5$. However, not all responses of homogeneous systems to variation in resource abundance generalise to the distinct forager system. For example, there is an additional (local, not global) maximia in the value of $w_5^{(3)}$ for the homogeneous system (see Figures 4b and 6f.ii), which is localised around $D=1$ in Figure 6f.ii, and is therefore unique to groups with similarly skilled foragers. This suggests that there may be qualitative differences in how groups with homogeneous and non-homogeneous knowledge (acting under optimal collective processing schemes) may respond to the availability of resources in the environment. In particular, heterogeneous groups may distribute knowledge between subgroups of a different size to that of homogeneous groups, represented here through the different orders of space sharing observed.

The shift in behaviour which was observed at $D=3$ in the resource-abundant scenario does not persist in the resource-constrained scenario, with the local maxima in both *K* and $C_1$ (which characterised this shift in behaviour) disappearing at intermediate resource constraints (compare Figures 6c and 6d to Figures 5b and 5c). Therefore in animal systems the socio-spatial impact of distinct foragers upon the group may be less significant in resource-constrained scenarios, as the effective benefit of having an increased foraging ability is reduced.

## Heterogeneous Knowledge Scenario

In this scenario we consider the impact of general heterogeneity in individual capacities for knowledge upon the optimal spatial structure of the group. The amount of heterogeneity is described through variation in the parameter $\sigma$, as outlined in Table 2. We again differentiate between resource-abundant and resource-constrained scenarios.

### Resource-Abundant Environment

**Fig. 7:**
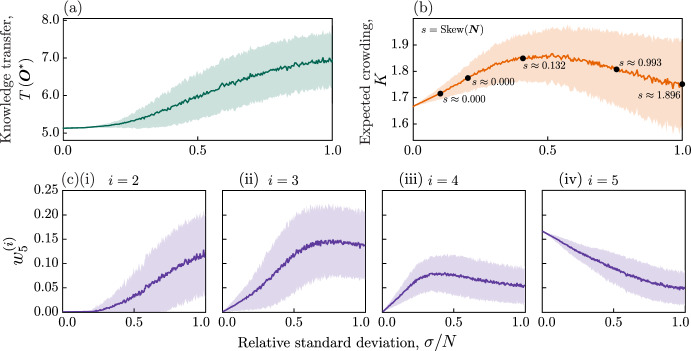
Results from our general heterogeneity scenario in the resource-abundant case with mean foraging ability $N=10^4$, $\sigma \in [0, N]$ and group size $n=5$. Specific values of $\sigma$ are obtained from 250 uniformly spaced points in this interval. Shown are: (a) the value of the objective function at the optimal solution, $T(\boldsymbol{O}^*)$, (b) the expected number of individuals with knowledge of each point under the optimal structure, *K*, with black dots showing the skewness values of the underlying distribution for $N_i$, denoted *s*, for the corresponding $\sigma$ value, (c) the relative orders of spatial overlap, $w_5^{(i)}$, for (i) $i=2$, (ii) $i=3$, (iii) $i=4$ and (iv) $i=5$. In each plot, solid lines are the average value across each of the 500 Monte Carlo simulations for each value of $\sigma$, and shaded regions show one standard deviation from this point. We assume that $F>F^*$, so we can neglect the foraging constraint for each value of $\sigma$ (color figure online)

As the heterogeneity in foraging abilities (represented by $\sigma$) is initially increased, there is a steady increase in both the group-level knowledge transfer, *T* (Figure 7a), and the expected point knowledge *K* (Figure 7b). This can be explained by our results from Section 4, where we saw that increasing or decreasing the ability of one individual gives rise to increasing *T* and *K*, where ‘better’ foragers increase their space sharing to support other individuals and ‘worse’ foragers increase their space sharing to gain support from other individuals, with both of these effects contributing positively to the group-level knowledge transfer (but yielding differing individual-level costs to each kind of forager). With the heterogeneous scenario, increasing $\sigma$ leads to the more likely introduction of highly skilled and lowly skilled individuals, in turn increasing $T(\boldsymbol{O}^*)$. With this scenario, *K* reaches lower maximum values and varies less overall when compared to the distinct forager scenario, because the presence of more skilled foragers is balanced by the presence of less skilled foragers. As $\sigma$ continues to increase, the distribution of $N_i$ values becomes wider, but due to the truncation at 0, will eventually only widen in the positive direction, increasing the skewness of the distribution, leading to ‘better’ foragers having more knowledge without changing the knowledge of the ‘worse’ foragers. Increasing $\sigma$ beyond this point is therefore similar to increasing *D* in the previous scenario. The increase in skewness leads to the value of *K* eventually decreasing, as the small number of highly skilled individuals have an increasingly high proportion of unique knowledge^3^. Meanwhile $T(\boldsymbol{O}^*)$ continues to increase, as the best forager (who is likely to be substantially better than the rest of the group when they exist, due to the positive skewness of the underlying distribution) provides increasing knowledge to the rest of the group (although this forager is unlikely to benefit from the spatial organisation at high $\sigma$).

To consider how these results depend upon the choice of distribution, we considered a modification to this scenario where $N_i$ is distributed according to a log-normal distribution (for details, see Section S1.4), where the skewness is consistently higher, and the probability density around $N_i=0$ is much lower (so both the ‘better’ and ‘worse’ foragers have improved foraging abilities, while the mean foraging ability remains fixed). With this modification, all measures of the spatial structure had qualitatively similar behaviours when increasing $\sigma$ (Figure S3), but we observed consistently lower values of $T(\boldsymbol{O}^*)$ (Figure S3a), suggesting that the support of ‘worse’ foragers contributes more to the group level knowledge transfer than the ‘better foragers’ providing additional support to the group knowledge. This is expected because, as we saw in the previous scenario, increased foraging ability provides diminishing returns to the objective value (Figure 5d) as the increased knowledge is balanced by the decreased opportunities for sharing with remaining group members.

Also with increasing $\sigma$, the proportion of highest-order space sharing, $w_n^{(n)}$, falls and there is a rise in the proportion of each lower-order sharing, $w_n^{(i)}$ for all $i=2,\dots , n-1$ (Figure 7c). This suggests that the optimal structures which are most centralised, where there is greatest alignment with the information centre hypothesis, appear in groups with homogeneous foraging abilities (in an unconstrained foraging environment). Ecologically, this implies that homogeneous groups may exhibit greater group cohesion whenever they are organised for optimal collective knowledge processing as all individuals have intersection in their core areas. This may facilitate the emergence of other group social traits related to high group attendance, such as common sleeping areas.

### Resource-Constrained Environment

**Fig. 8:**
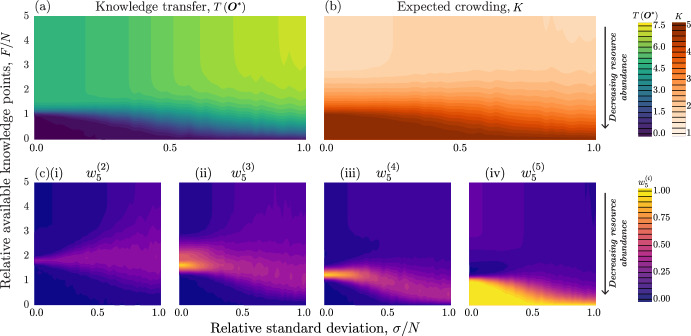
Results from our general heterogeneity scenario with mean foraging ability $N=10^4$, $\sigma \in [0, N]$, foraging constraint varying between $F\in [0.01, 4\times 10^4]$ and for group size $n=5$. Specific values of $\sigma$ and *F* are obtained from 50 uniformly spaced points over their respective intervals, with each *F* rounded upwards so $F\in \mathbb {N}$. Shown are: (a) the value of the objective function at the optimal solution, $T(\boldsymbol{O}^*)$, (b) the expected number of individuals with knowledge of each point under the optimal structure, *K*, (c) the relative orders of spatial overlap $w_5^{(i)}$ for (i) $i=2$, (ii) $i=3$, (iii) $i=4$ and (iv) $i=5$. In each contour plot, the average values across each of the 500 Monte Carlo simulations for the corresponding value of $\sigma$ and *F* are shown (color figure online)

When including resource constraints, we find that extent to which differences in foraging ability can lead to variation in optimal network structures is reduced, as the effective knowledge of the best forager is now limited (Figure 8). As $\sigma$ increases, the value of $T(\boldsymbol{O}^*)$ increases until the constraint is extremely tight (until *F* approaches 0; see Figure 8a). This is because with higher values of $\sigma$ there are more individuals with a very low knowledge capacity who are not restricted in their effective knowledge even at low *F* values, resulting in non-trivial spatial structures where a more homogeneous group would have a trivial structure (where $w_n^{(n)}=1$). This is evidenced by the fact that when knowledge values are drawn from a log-normal distribution (where the ‘worst foragers’ typically have higher knowledge), there is a much wider region of *F* values which consistently yield these trivial structures (compare Figure 8a with Figure S4a). On the other hand, the pattern of *K* initially increasing and then decreasing with $\sigma$ disappears when including resource constraints, for both distributions considered (Figures 8b and S4b). These results collectively help to corroborate our claim that variation in $T(\boldsymbol{O}^*)$ and *K* with $\sigma$ can be accredited to distributional skewness, which is limited whenever there are foraging constraints, as there is now an upper (*F*) and lower (1) bound on $N_i$.

We continue to observe patterns of interaction order selection with *F* in this scenario, as in Sections 3.2 and 4.2. Particularly, for each $\sigma$ value, there are peaks in the values of $w_n^{(i)}$, where *i* is decreasing sequentially with *F* (and this pattern persists when normalising $\boldsymbol{N}$). This is represented in Figure 8c by the horizontal streaks of maximal values of $w_n^{(i)}$. When initially increasing $\sigma$ from 0 there is no variation in the value of *F* at which these maximal values occur, implying that resource availability can select for a specific order of interaction in a diversity of systems. Furthermore, these ‘streaks’ of higher values in the heterogeneous population only reach global maximality (i.e. $w_n^{(i)}=1$) for small values of $\sigma$, when abilities are more homogeneous. Therefore, more uniform/ordered spatial structures (where the order of interactions is fixed throughout) may be a unique property in the optimal spatial structure of groups with more homogeneous information gathering capacities. As $\sigma$ increases further, these peaks reduce in their height and widen across the *F* axis (most prominently for lower-order interactions), with the peak starting to appear at lower values of *F* (most prominently for higher-order interactions). The proportion of lowest-order (dyadic) space sharing is consistently non-decreasing with $\sigma$, and there is an especially wide dispersion of values at higher $\sigma$, suggesting that heterogeneity promotes lower order interactions across most environments. These results highlight how within-group variation in foraging abilities may be a key driver of spatial complexity in a variety of natural systems, and suggests that the movement algorithms of artificial systems should be designed with the amount of device heterogeneity (e.g. in movement capabilities, storage capacities) explicitly accounted for.

We additionally find that some intermediate values of *F* reverse the relationship found in the resource-abundant case: the proportion of highest-order sharing, $w_n^{(n)}$, can increase with $\sigma$, while the proportion of lower-order sharing, $w_n^{(i)}$, can decrease for $i=2,\dots , n-1$ (for example, around $F=1.9N$ in Figure 8c). Therefore, some groups with large variation in individual information capacities can have an optimal spatial structure which is centralised and therefore aligning with the information centre hypothesis, if placed in an environment with particular resource constraints.

## Discussion

Our model yielded important insights into how collectives can organise their spatial structures to promote optimal sharing of knowledge, which is of strong relevance across domains in heterogeneous environments. Our three scenarios explored how the optimal spatial structure of a collective foraging group may vary according to differences in individual abilities, and how these structures may respond differently to variation in the resource abundance of their environment.

One key finding was that the distribution of knowledge in optimally structured groups was heavily influenced by the level of resource availability (represented in this study through the parameter *F*). Particularly, we consistently observed that the value of *F* promoted specific orders of space sharing in the optimal structure of the group, which may be interpreted as certain subgroup sizes. In both lightly and heavily constrained environments our results suggested that we would expect commonly-shared foraging points within the core areas of all group members, in line with the information centre hypothesis (Evans et al. 2016) for ecological systems. However, systems which are heterogeneous in their information gathering abilities or placed in environments with intermediate resource levels were found to instead have their optimal spatial structures dominated by lower-order spatial overlaps. This implies that we may expect a relatively worse support for the information centre hypothesis in such systems and a better support for other, less centralized foraging strategies. If we interpret prominence of lower-order interactions as indicative of a fission-fusion type society (primarily observed in heterogeneous environments (Aureli et al. 2008; Dunbar 1988) and characterised by reduced group cohesion and high fluidity in subgroup composition (Couzin and Laidre 2009)), then our model suggests that intermediate resource availability or heterogeneity in foraging abilities can give rise to stronger fission-fusion dynamics. This result aligns with previous theory relating fission-fusion dynamics and foraging behaviour (Ramos-Fernandez et al. 2006).

Using specific orders of spatial interaction allowed collectives to maintain high levels of knowledge sharing in constrained environments, but this organisation caused each individual to maintain fewer uniquely known foraging points (quantified here by $C_k$), potentially representing an individual-level cost of the spatial organisation of the collective (Lee et al. 2016). These potential costs were higher in larger groups (for fixed levels of resource abundance, *F*), with larger groups also exhibiting more crowding around foraging sites (which, in animal foraging systems, may devalue the knowledge of these points by increasing competition). This has implications in the evolutionary ecology of group size. In particular, in sufficiently resource abundant environments, we observed quadratic-like growth in the objective function value with group size, *n*, such that an increase in group size translates to a linear benefit to each individual (*on average*, since benefits could be spread unequally across the group). Resource availability of the environment might then select for a particular, intermediate group size (in agreement with pre-existing theory; Seiler and Robbins (2020); Gibbs et al. (1987)), by determining the individual-level costs to a particular structure compared to the increased benefit from the group-level (both of which should theoretically increase with group size). This also aligns with the theory that intermediately sized groups have energetically optimal space-use strategies (Markham et al. 2015). In our non-homogeneous scenarios, we saw that the addition of a Forager with high foraging knowledge capacity can improve the collective information processing of the group. However, this group-level benefit is unequally spread between group members, with the better forager taking higher costs in the spatial structure than other individuals (engaging in additional sharing). These effective foragers may also be less likely to get involved in information sharing behaviours in the first place, as the advantages of reciprocity from the relatively worse foragers may be relatively small. This represents another clear conflict between individual-level and group-level effects of the optimal spatial organisation, which again may help promote particular group size or may select for the amount of within-group heterogeneity in an ecological context. In artificial systems, these individual-level costs could represent the financial costs of a single agent/robot (Schroeder et al. 2019), and expected resource availability could therefore help to inform budgeting strategies in collective systems design. Further analytical work in this framework could help bypass computational issues regarding dimensionality, and yield a more precise synthesis of the role of group size in collective intelligence systems.

Our model framework is general and is designed to be applicable to a broad range of collective systems. There are still a variety of natural extensions to this model which may increase applicability. It would be straightforward to consider different forms of variation in $\boldsymbol{N}$. Considering distributions with varying degrees of skewness and kurtosis could improve our mechanistic understanding of the relationship between heterogeneity and optimal spatial structure in this context. Different distributions could also allow the model to tackle more specific ecological questions. For example, we could consider multi-modal distributions to consider the impact of differing social roles, where certain individuals may be obliged to engage in more foraging behaviour than others (for example, using a bi-modal distribution to represent differing male/female roles). Another interesting direction would be the inclusion of non-independent movement of individuals. Ecologically, the assumption of independence implies that we are modelling a system where either group cohesiveness has not yet formed, where information gathering is an inherently solitary task, or where there is high de-synchronization of needs and movement motivations (e.g. systems with high fission-fusion dynamics and low predation pressure; Aureli et al. (2008)). By explicitly tracking each potential foraging point, one could implement following behaviours, which are well-established empirically (Palacios-Romo et al. 2019). A similar system to this optimisation framework but with an agent-based framing could allow the model to represent and compare different movement algorithms for robot swarms (Tarapore et al. 2020) in variable environments and consider variation in the number of individuals - possibly helping to inform systems design in novel applications of swarm technology. There is a rich variety of other possible future directions, including: heterogeneity in resource values, explicit temporal variation in resource availability and multi-level optimisation, which may all help to further explain the dramatic variation in spatial structures observed in nature and help to inform collective systems design.

## Conclusion

Collectively, our paper highlights how resource availability and variation in foraging capacities can influence the optimal spatial structures of collective foraging or information gathering systems. Given the rising prevalence of robot swarms in human applications, and rapid global change driving variation in food abundance for animal systems, such understanding could shape much needed theoretical and empirical research across diverse contexts. Our work, therefore, exemplifies the importance of considering higher-order spatial interactions in studies of natural and artificial collectives. Further examination of higher-order spatial structure could generate insights of broad ecological relevance (e.g. animal colonies), with potential applications for the bio-inspired design of artificial collective systems (e.g. swarm robotics systems).

**Supplementary information**

Supplementary material available has been submitted alongside this article.

## Data Availability

There is no data associated to this manuscript.

## Footnotes

1 For brevity throughout the text, we write $w_n^{(i)}=w_n^{(i)}(\boldsymbol{O})$, $K=K(\boldsymbol{O})$ and $C_k = C_k(\boldsymbol{O})$

2 We consider the case where *N* is even throughout this paper, emphasising that the results are qualitatively identical in the odd case.

3 This is indicated by the switch to a decreasing relationship between *K* and $\sigma$ occurring around about the point at which the skewness begins to increase (compare Figure 7b to Figure S2).

## Code Availability

The underlying code for this study is available at: https://github.com/rswalker-eco/Spatial_topological_optimisation.git.
